# Application of Baculovirus Expression Vector System (BEVS) in Vaccine Development

**DOI:** 10.3390/vaccines11071218

**Published:** 2023-07-08

**Authors:** Qiaonan Hong, Jian Liu, Yuquan Wei, Xiawei Wei

**Affiliations:** Department of Biotherapy, Laboratory of Aging Research and Cancer Drug Target, State Key Laboratory of Biotherapy, National Clinical Research Center for Geriatrics, West China Hospital, Sichuan University, No. 17, Block 3, Southern Renmin Road, Chengdu 610041, China

**Keywords:** baculovirus, insect cell, BEVS, vaccine, clinical application

## Abstract

Vaccination is one of the most effective strategies to control epidemics. With the deepening of people’s awareness of vaccination, there is a high demand for vaccination. Hence, a flexible, rapid, and cost-effective vaccine platform is urgently needed. The baculovirus expression vector system (BEVS) has emerged as a promising technology for vaccine production due to its high safety, rapid production, flexible product design, and scalability. In this review, we introduced the development history of BEVS and the procedures for preparing recombinant protein vaccines using the BEVS platform and summarized the features and limitations of this platform. Furthermore, we highlighted the progress of the BEVS platform-related research, especially in the field of vaccine. Finally, we provided a new prospect for BEVS in future vaccine manufacturing, which may pave the way for future BEVS-derived vaccine development.

## 1. Introduction

**Figure 1:**
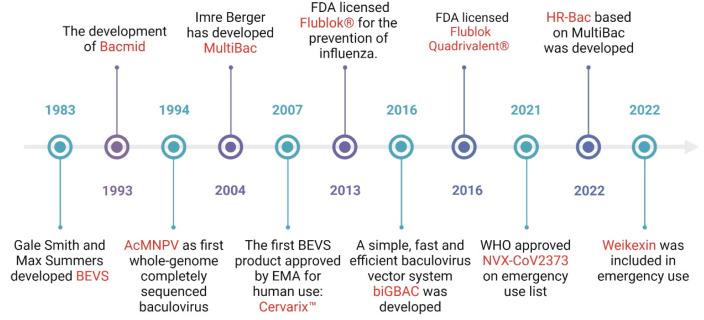
A timeline of crucial development of the BEVS. BEVS was developed initially in 1983 and then gradually advanced to the present state of development. The timeline mainly illustrates two aspects: the development of BEVS and its application in vaccine development.

With the expansion of the global vaccine market caused by COVID-19, the baculovirus expression vector system (BEVS) has gradually attracted the attention of vaccine producers. BEVS is a well-established platform for exogenous protein expression that has been used effectively for vaccine production, gene therapy, and other applications for several decades [1]. Initially developed in the 1970s to control agricultural pests, the BEVS has evolved into an essential platform for producing various recombinant proteins, including those used in vaccines [2]. In 1983, BEVS was first used as a recombinant baculovirus to produce heterologous human IFN-β [3]. The regulatory acceptance of BEVS in manufacturing human vaccines was a critical milestone, with the approval of Cervarix™, the first BEVS-derived vaccine against cervical cancer, in 2007 [4]. Subsequently, in 2013, a BEVS-derived vaccine against influenza was approved by the FDA [5]. The timeline for the development of the BEVS platform is presented in Figure 1. Currently, more than ten BEVS-derived vaccines are available, including but not limited to Cervarix™ (GSK, London, UK) against cervical cancer, Flublok^®^ (Sanofi Pasteur, Paris, France) and Flublok Quadrivalent^®^ (Sanofi Pasteur, Paris, France) against influenza, and NVX-CoV2373 (Novavax, Malvern, PA, USA) and Weikexin (Westvac, Chengdu, China) against COVID-19, proving that BEVS has become a promising vaccine production technology [6].

Compared to other vaccine production platforms, the BEVS platform offers several advantages for vaccine production, including its ability to rapidly produce scale quantities of protein, its capacity to introduce post-translational modifications, and its high safety and regulatory compliance [7]. These advantages, coupled with their successful application in vaccines, have garnered the interest of academic researchers and industry leaders. They are currently using BEVS to develop next-generation vaccines, gene therapy vectors, and other complex biopharmaceutical proteins [8]. Nonetheless, the BEVS platform has some limitations, including unstable expression and inappropriate protein glycosylation, which have been addressed in recent years with the development of molecular biology by optimizing the viral vector components and editing the engineered cell lines [9,10].

With the approval of new products of BEVS, such as influenza and SARS-CoV-2 vaccines, the application of this platform in vaccine development will be further expanded. This review covers the development history of BEVS and BEVS’s general recombinant protein vaccine production process. Furthermore, we also summarize the platform’s advantages, limitations, and research progress on optimizing the BEVS. Finally, this review also highlights the progress of BEVS applications in vaccines, particularly in SARS-CoV-2 vaccines. In addition to summarizing the latest research progress and applications of BEVS as a vaccine research platform, this review discusses how to improve strategies to further develop the BEVS vaccine research platform and manufacture vaccines with lower cost and higher quality, thus serving as a reference for developing next-generation vaccines.

## 2. Composition and Workflow of BEVS

The fundamental feature of the baculovirus expression vector system (BEVS) is to replace the non-essential gene in the baculovirus with the foreign gene of interest, use the baculovirus as the foreign gene carrier, and use the insect cell as the host for gene amplification and target protein expression. The BEVS consists of three parts: a transfer plasmid, a baculovirus vector, and an insect host cell line [11]. The transfer plasmid contains foreign genes to be transferred into the baculovirus. Baculovirus is an enveloped, double-stranded DNA virus that specifically infects arthropods in nature and belongs to the Baculoviridae family [12]. Autographa californica multicapsid nucleopolyhedrovirus (AcMNPV) and Bombyx mori nucleopolyhedrovirus (BmNPV) are the most commonly used baculovirus vectors [13]. AcMNPV, the first baculovirus with complete sequencing, is widely used as a research model [14]. A significant advancement in the field was the development of bacmid, a vector containing the entire AcMNPV genome that can be propagated in *E. coli* cells, which was developed into the commercialized Bac-to-Bac system we know today. The two most common cell lines used in BEVS for commercial or academic purposes are the Sf9 and Hi5. In general, Sf9 is more suitable for virus amplification and packaging, and Hi5 is more suitable for expressing secreted proteins [15].

**Figure 2:**
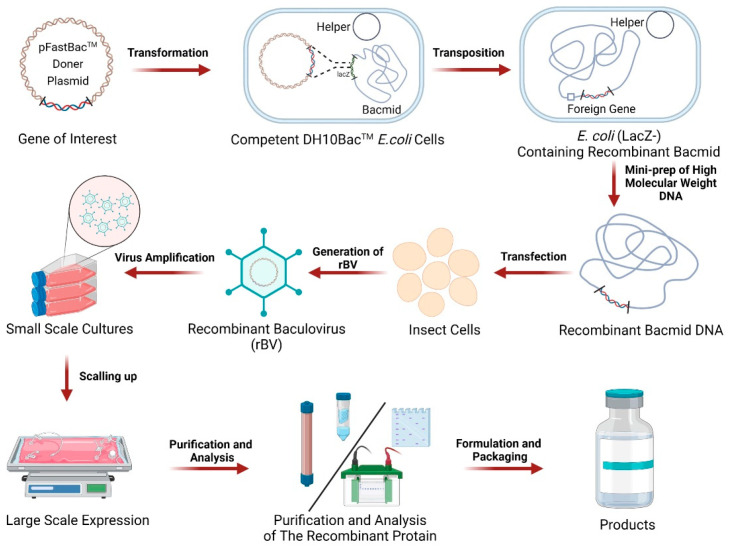
A flow chart of recombinant protein vaccine production using BEVS. The gene of interest in the donor plasmid encoding the protein is transformed into a uniquely competent *E. coli* containing bacmid and helper plasmid. Translocations occur in *E. coli*, where the foreign gene is transferred from the donor plasmid to the bacmid. Bacmid is then extracted from *E. coli* and used to transfect insect cells to produce recombinant baculovirus (rBV). The rBV is amplified to obtain a high-titer virus for the large-scale expression of proteins. After purification and analysis, the recombinant protein is formulated and packaged into products.

Since baculovirus was first used to produce heterologous human IFN-β protein in insect cells, the BEVS platform has been widely used to produce a variety of other heterologous proteins, including surface-displayed proteins/antigen carriers, virus-like particles (VLPs), heterologous virus vectors, and gene delivery vehicles [16]. As illustrated in Figure 2, preparing recombinant protein vaccines using the BEVS platform mainly involves obtaining bacmid containing the target gene, recombinant baculovirus production, and protein purification. First, the donor plasmid encoding the antigenic protein is used to transform a uniquely competent *E. coli* (containing bacmid and helper). Translocations occur in *E. coli*, where genes encoding antigenic proteins are transferred from a donor plasmid to the bacmid. The bacmid is then extracted from *E. coli* and used to transfect insect cells to produce recombinant baculovirus. The recombinant baculovirus needs to be further amplified to obtain a high-titer recombinant baculovirus for large-scale production of recombinant proteins. High-titer recombinant baculovirus is added to the insect cells in their logarithmic growth phase. Typically, cells or supernatants are harvested 48–96 h post-infection and used to purify proteins. Large-scale protein production is usually carried out in bioreactors. Finally, the protein is further purified and analyzed before releasing the final product.

## 3. Advantages and Limitations of BEVS

The baculovirus expression vector system (BEVS) platform offers many advantages, including manufacturing speed, flexible product design, high safety, and scalability. The many advantages of the BEVS platform make it one of the leading platforms for the preparation of recombinant protein subunit vaccines, but it is not suitable for all products. Factors such as post-translational modifications and protein properties should be considered when selecting a manufacturing platform.

The BEVS platform has the advantages of faster manufacturing speed and greater scalability compared with traditional vaccine preparation. For example, in the case of influenza, which occurs almost annually, producing an egg-adapted, high-yielding seed virus is a slow process, taking an average of six months to manufacture a traditional egg-based influenza vaccine [17]. Consequently, the available influenza vaccine often does not match the circulating strain [18]. In contrast, the BEVS-derived influenza vaccine can be manufactured in just one and a half months, allowing for a faster response to new influenza outbreaks [19]. In addition, cost-effectiveness is critical for the successful commercialization of vaccines, especially animal vaccines. BEVS has two characteristics that make it easy to manufacture on a large scale at a lower cost. First, the substrates are insect cells, which can grow in an inexpensive animal product-free medium to high densities [20]. For instance, expresSF+^®^ cells produce recombinant proteins at scales ranging from 2 to 21,000 L [21]. Second, when optimized for large scales and multiple passages, insect cells, and baculovirus can be cultured in bioreactors of any size, limiting the culture size only to the bioreactor size [21]. The scalability, speed of manufacture, and existing manufacturing capacity of the BEVS platform make it appealing for commercial manufacturing.

Another advantage is the flexible product design. The BEVS platform can accommodate the insertion of larger fragments of foreign DNA and perform some post-translational processing modifications on the expression products (with exceptions; see below). The system can also express multiple foreign proteins simultaneously, providing a new idea for studying multiple protein interactions and assembly mechanisms. For instance, The MultiBac system based on the BEVS platform is suitable for simultaneously expressing multiple proteins, which can generate specific multi-subunit protein complexes [22]. HR-Bac is another MultiBac-based design that facilitates expression screening and potential high-throughput applications [23]. In addition, BEVS provides appropriate post-translational modifications such as phosphorylation, glycosylation, ubiquitination, and acetylation, which are used in protein function and crystallographic studies [24,25].

Safety is the primary consideration for vaccines since many healthy people, including children, are vaccinated. The high safety of baculovirus is that it only infects insect cells, is generally considered a safe biological pesticide, and does not infect vertebrates [26]. The baculovirus cannot integrate its DNA into the host genome without selection pressure, making it biologically safe for humans [27]. Furthermore, safety tests conducted by the Organization of Economic Co-operation and Development in 2002 and the European Commission’s Health and Consumer Protection Directorate-General in 2008 found that baculovirus has no adverse effects on human health and is not carcinogenic, genotoxic, or pathogenic in mammalian cells [28]. The baculovirus allows a single cell line to express different proteins and eliminates the need to repeatedly qualify a cell line to ensure purity and safety, while maintaining high productivity [8]. Additionally, BEVS does not require handling live or potentially dangerous pathogens and does not necessitate costly biocontainment measures. The recombinant products prepared using BEVS are free of pathogens, eggs, and the most potentially harmful or allergenic chemicals, ensuring their purity [29].

The BEVS platform has unique advantages that make it an attractive option for producing many biologics; however, several limitations should also be considered. A typical example of post-translational modification is glycosylation. Although BEVS can produce protein N-glycosylation, it is not equivalent to that found in higher eukaryotes [30]. Mammalian cells produce proteins with complex-type N-glycans that have terminal sialic acids. In contrast, insect cells produce proteins with paucimannose-type N-glycans at the same sites as the complex glycans, resulting in simple side chains [30,31]. Thus, although insect cells offer many post-translational modifications, proteins requiring complex post-translational modifications and folding are best produced in mammalian expression systems. In addition, for protein properties, it is best to prepare some proteins that do not require post-translational modification or simple post-translational modified proteins in *E. coli* or yeast cells, which can quickly produce high-yield proteins at low cost [32].

## 4. Strategies for Optimizing BEVS

In recent decades, researchers have extensively studied several optimization strategies to address the limitations of the baculovirus expression vector system (BEVS), which can be classified into two categories. The first strategy is the engineering of a baculovirus vector. Replacing the non-essential baculovirus gene polyhedrin coding sequence with the nucleotide sequence of interest is the core of BEVS technology. Baculovirus genetic engineering optimizes baculovirus vector components to improve protein expression. The second strategy is the engineering of the cellular host to delay the death of insect cells after baculovirus infection, which is a critical factor in limiting protein expression. For example, genetically engineered insect cell lines or RNA interference (RNAi) with apoptotic genes delay cell death. Several representative optimization strategies for BEVS are shown in Table 1.

### 4.1. Engineering of the Baculovirus Vector

Engineering of the baculovirus vector includes the optimization of vector elements and baculovirus genetic engineering. The expression of recombinant proteins is regulated by DNA regulatory elements in the regions near the gene of interest. The optimization of vector elements, including promoters and enhancers, can improve the level of protein expression. In 2016, Martínez-Solís et al. demonstrated that the orf46 promoter had an additive effect when combined with pH or p10 promoters [33,34]. Other early promoters, such as 39k or gp64, have been shown to contribute to recombinant protein expression during the early stages of cell infection [34]. Additionally, Sugai et al. found that introducing the polar amino acid asparagine into the C-terminal of the SP1 region can enhance the secretion of recombinant silkworm protein. This approach can also be applied to the BmN, Sf, and Tn cell lines [35]. Immediate early promoters, such as OpIE2 and IE1, have been identified as effective initiators of foreign gene expression in insect cells using plasmid-based or piggy Bac-based vectors [36,37].

In 2018, Lee et al. discovered that enhancers including hr5, burst sequences (BSS), and vp39 could enhance the activation ability of the pH promoter [38]. Tiwari et al. also found that the protein expression in insect cells is higher when the homologous region 1 (hr1) is present than when it is absent [39]. Furthermore, the p19 protein acts as an RNAi inhibitor and can increase the expression of downstream pH and OpIE2 promoters as enhancers, significantly improving baculovirus production [40].

Genetic engineering of baculovirus can be achieved by removing non-essential genes for in vitro viral replication or adding genes beneficial to the viral genome to enhance protein expression. For example, during the late stages of baculovirus infection, the insect host’s secretory pathways are interrupted, leading to unique terminal liquefaction [41]. In this case, the gene deletion strategy can improve baculovirus and enhance recombinant protein production, for example, by deleting the unnecessary genes p10 and p24. To improve secretion function and reduce the possibility of recombinant protein degradation, baculovirus vectors that lack chitinase and cathepsin have been designed, as chitinase interacts with cathepsin after a large amount of accumulation.

Another approach is the introduction of anti-apoptotic genes such as P35. For example, Sf9 cells stably expressing the P35 protein can resist apoptosis and produce higher levels of recombinant proteins. Based on these principles, Dong et al. developed a vector named the “multiple editing anti-BmNPV therapeutic complex CRISPR-Cas9 system, PSL1180-Cas9-sgIE1-sgLEF11-sgGP64 (sgMultiple)”, which is beneficial for antiviral therapy. This vector can effectively regulate various genetic editing pathways and disrupt the replication of BmNPV [42].

### 4.2. Engineering of the Cellular Host

Engineering of the cellular host includes RNAi technology and establishing engineering cell lines. BEVS is a transient expression system in which baculovirus infection induces programmed cell death of insect cells 3–4 days post-infection, followed by terminating the protein expression. Hebert et al. demonstrated that infected cells showed signs of apoptosis 48 to 72 h after infection, with significantly increased caspase activity [43,44]. Previous studies have shown that inhibiting cell apoptosis effectively increases protein production [45]. RNAi technology is a powerful and efficient method for manipulating gene expression in insect cell lines, offering versatility to silence specific genes. RNAi-mediated apoptosis-related gene silencing is an excellent example of how RNAi technology can improve the BEVS platform. While apoptosis is a necessary physiological function to eliminate insect cells, it can also affect the quality and yield of recombinant products [46]. Chen et al. extended the expression period of recombinant protein to 5 days by inhibiting the expression of caspase-1, the last enzyme in the apoptosis pathway of insects, so that the protein production reached more than 400 mg/L [45]. Furthermore, several research groups have established cell lines derived from Hi5, BmN, and Sf9, which can stably downregulate caspase-1 expression via RNAi, prolonging the production time of the culture and increasing the yield of recombinant proteins [43,44,47,48].

Currently, another limitation of using insect cell lines for recombinant protein production is that they provide fewer complex N-glycans than recombinant protein produced by mammalian cell lines. Several engineering cell lines have been developed to overcome this limitation. The commercial transgenic mimic Sf9 (SfSWT-1) insect cell line is one such cell line. After modification, this cell line was derived from Sf9 cells. It can produce highly processed recombinant proteins with terminal sialic acid complex-type N-glycans. The recombinant protein glycosylation level in mimic Sf9 is higher than that in Sf9 or Hi5 cells because it integrates with five mammalian glycosyltransferases [49]. Toth et al. established an efficient transgenic insect cell line (Sf39kSWT), which was developed by stably transforming Sf9 insect cells with a group of nine mammalian glycogen under the control of 39k-inducible promoters, and the expression of its foreign gene did not affect the growth and stability of transgenic insect cell line [50,51]. The CRISPR-Cas9 technique developed a novel derived Sf9 insect cell line (SfFDLt1), which was otherwise unable to produce a fused lobed (FDL) protein, an enzyme that antagonizes N-glycan elongation and enhances insect glycosylation capability [52].

## 5. BEVS-Derived Vaccines

### 5.1. BEVS-Derived Commercial Vaccines

Currently, thirteen BEVS-derived vaccines have been approved, including eight vaccines for human use and five for veterinary use; details are provided in Table 2. A successful representation of BEVS-derived subunit vaccines is Flublok^®^. The approval of Flublok^®^ in 2013 improved the stagnation of the influenza vaccine manufacturing technology. Flublok^®^ is a recombinant trivalent hemagglutinin (rHA) vaccine for seasonal influenza developed using the baculovirus expression vector system (BEVS) platform [53]. Hemagglutinin (HA) is a dominant glycoprotein found on the surface of the influenza virus and is considered a critical antigen for recombinant influenza vaccines. Flublok^®^ includes the HA protein antigen derived from three strains of influenza virus included in the World Health Organization’s annual influenza vaccine, which is updated annually. Compared to traditional trivalent inactivated influenza vaccines, the most significant advantage of Flublok^®^ is its short production cycle for large-scale production, which can keep up with the continuous variation of the influenza virus each year. Clinical results have shown that Flublok^®^ meets the FDA’s standards and is well-tolerated and immunogenic in over 3000 adults aged 18 and over [54]. It has an excellent protective effect against the influenza virus, especially in adults over 65 and high-risk groups, such as immunocompromised individuals [20]. In addition, Flublok Quadrivalent^®^, a quadrivalent recombinant influenza vaccine (RIV4) manufactured by Sanofi Pasteur, is also available. Compared to egg-grown quadrivalent-inactivated influenza vaccines (IIV4), Flublok Quadrivalent^®^ demonstrates comparable immunogenicity, with slight local and systemic reactions [55].

Cervarix™ is a successful representative of the VLP vaccine derived from BEVS developed by GSK. HPV is responsible for approximately 500,000 new cervical cancer cases yearly. Cervarix™, a bivalent vaccine manufactured by GSK and produced using the BEVS platform, is an effective HPV prophylactic vaccine that primarily targets HPV-16 and HPV-18. Cervarix™ protects for at least 6.4 years, and high levels of total and neutralizing antibodies against HPV-16 and HPV-18 can be detected up to 7.3 years after vaccination [5]. Compared with other HPV vaccines, such as Gardasil^®^ produced using yeast expression systems and Cecolin^®^ produced using *E. coli* expression systems, Cervarix™ induces significantly higher levels of neutralizing antibodies against HPV-16 and HPV-18, as well as increased frequency of antigen-specific memory B cells and T cells [5].

Another representative BEVS-derived vaccine is the SARS-CoV-2 vaccine approved for emergency use, including Novavax’s NVX-CoV2373, Westvac’s Weikexin and its trivalent vaccine, Sanofi/GSK’s VidPrevtyn Beta (Sanofi/GSK, Paris, France/London, UK), and Vaxine/CinnaGen Co.’s SpikoGen^®^ (Vaxine/CinnaGen Co., Adelaide, Australia/Tehran, Iran) [56]. NVX-CoV2373 is a protein subunit vaccine for SARS-CoV-2, which comprises recombinant full-length S protein expressed in Sf9 cell lines as the antigen and Matrix-M as the adjuvant [57]. NVX-CoV2373 is a low dose of NVX-CoV2373 and Matrix-M is highly immunogenic in baboons. They trigger high titers of anti-S and functional antibodies, blocking S protein binding to human angiotensin-converting enzyme 2 (hACE2), neutralizing viral infections, and inducing antigen-specific T cells [58]. In phase III of the clinical trials, it was found that the protection rate of two doses of NVX-CoV2373 vaccine for adults against the SARS-CoV-2 infection was 89.7%, especially for the B.1.1.7 variant [57]. The Weikexin vaccine induces potent functional antibody responses in immunized mice, rabbits, and non-human primates (*Macaca mulatta*) as early as 7 or 14 days after a single dose. It can neutralize the infection of SARS-CoV-2 pseudovirus and live SARS-CoV-2 in vitro, protecting non-human primates against the attack of SARS-CoV-2 in vivo [59]. Weikexin’s trivalent SARS-CoV-2 trimer protein vaccine is the world’s first SARS-CoV-2 vaccine approved for emergency use against XBB and other variants. Fourteen days after inoculation, the protective effect is 93.28% against infection with SARS-CoV-2 mutant strains, such as XBB.1, XBB.1.5, and XBB.1.9, with good safety. VidPrevtyn Beta is a preS dTM vaccine booster developed from parental strain D614 or variant B.1.351 (Beta). It was found that a single dose of the vaccine formulated as monovalent D614 (parental), B.1.351 (Beta), or bivalent (D614 + B.1.351) with AS03 adjuvant significantly boosted neutralizing antibodies produced by *Macaca mulatta* after the previous vaccination [60]. SpikoGen^®^ is also a protein subunit SARS-CoV-2 vaccine expressed in insect cell lines, comprising the recombinant S protein extracellular domain formulated with Advax-CpG55.2™ adjuvant [61].

In addition, BEVS has significantly contributed to veterinary vaccines. Two preventive vaccines against the classical swine fever virus and three preventive vaccines against porcine circovirus type 2 are currently available. Evaluation of the classical swine fever virus preventive vaccines, Porcilis^®^ Pesti (MSD Animal Health, Shanghai, China) and BAYOVAC CSF E2^®^ (Bayer AG/Pfizer Animal, Nordrhein-Westfalen, Germany/Groton, CT, USA), demonstrated that vaccinated pigs had better growth than the control groups, indicating that vaccination can effectively prevent the reproduction of the classical swine fever virus [62]. Similarly, preventive vaccines against porcine circovirus type 2, such as CircoFLEX^®^ (B. Ingelheim, Berlin, Germany), Circumvent^®^ PCV G2 (Merck Animal Health, Madison, NJ, USA), and Porcilis^®^ PCV (MSD Animal Health, Shanghai, China), have been shown to significantly increase antibody titers at 4 to 8 weeks following vaccination, leading to specific protective effects against the disease [63].

### 5.2. BEVS-Derived Clinical Vaccines

As a growing number of BEVS derivatives have been approved by global regulators and have entered the clinical phase, the public’s understanding of BEVS is becoming increasingly deeper. After four decades of development, the corresponding regulatory laws and regulations have been gradually improved, which has attracted interest from academic researchers to utilize BEVS to develop vaccines, and the public has become more aware of BEVS vaccines. Currently, BEVS has become one of the mainstream technologies in vaccine production.

#### 5.2.1. SARS-CoV-2 Vaccines in Clinical Trials

In addition to the vaccines already approved for marketing, many vaccine candidates produced via the BEVS platform are undergoing clinical trials, the details of which are provided in Table 3. The development of SARS-CoV-2 vaccines is a typical example. Protein subunit and VLPs vaccines have been developed using recombinant proteins produced via BEVS [64].

Protein subunit vaccines account for a large proportion of vaccine types in SARS-CoV-2, and the BEVS platform has the advantages of rapidity and flexibility to produce subunit vaccines. Subunit vaccines employ SARS-CoV-2 proteins, such as the spike (S) protein, which triggers the immune system. By modifying the S protein and replacing K986 and V987 in the S2 subunit with prolines, S2 is cleaved into a minute fragment, thus producing stable spike proteins in insect cells [65]. A further modification was carried out by inserting a C-terminal thrombin cleavage site and obtaining an “AGAG” sequence at the furin cleavage site, producing the recombinant protein S-2P [66]. This protein is a promising vaccine candidate that elicits highly neutralizing antibodies in monkeys, has significant reactivity in COVID-19 serum, and yields high amounts in insect cells. The S-2P protein became soluble after the T4 fold domain was replaced with the transmembrane domain. The new recombinant protein was a prefusion transmembrane-deleted spike (preS dTM) [67]. The preS dTM vaccine, adjuvanted with AS03 oil-in-water emulsion, can protect non-human primates from a high dose of SARS-CoV-2 infection. The preS dTM vaccine is currently in clinical phase II (NCT04762680).

VLPs vaccines are composed of multimer particles that mimic the spatial structure of natural virus particles. They contain hollow particles of one or more virus structural proteins but lack viral nucleic acid and are morphologically similar to authentic virus particles. The COVID-19 vaccine, synthesized using VLPs via the BEVS platform, involves three technologies. In the first technique, insect cells simultaneously express the E, M, and S proteins using triple-expression plasmids. Each component is self-assembled in the insect cells, which elicits a strong SARS-CoV-2 VLPs-specific humoral and cellular immune response in mice [68,69]. The second technique involves the co-transfection of a recombinant baculovirus expressing the full-length S, S1, or S2 protein with another recombinant baculovirus expressing influenza matrix protein 1 (M1) to form VLPs in insect cells. After immunization, mice exhibit higher levels of spike protein-specific IgG and its subclasses, with IgG2a being predominant [70]. The third technique employs the Spy Tag/Spy Catcher platform to couple the S1 protein to phage A205VLP nanoparticles to form an adjuvant-containing vaccine. After immunization, it was found to elicit an effective neutralizing antibody response to Wuhan and UK/B.1.1.7 variants and is currently in clinical phase I (NCT04839146) [71].

#### 5.2.2. Other Vaccines in Clinical Trials

In addition to SARS-CoV-2 vaccines, there are many BEVS-derived vaccines in the clinical trial phase. Norovirus is a major cause of acute gastroenteritis outbreaks and sporadic cases [72,73]. LigoCyte (Bozeman, MT, USA) and Baylor College of Medicine (Houston, TX, USA) have developed Norovirus VLPs using BEVS and are currently conducting clinical phase I and II trials (NCT00806962/NCT00973284), respectively. Clinical data have shown that a dose of 100 mg LigoCyte’s norovirus VLPs increased specific IgG and IgA antibody levels by 4.8-fold and 9.1-fold, respectively, compared with healthy people. In contrast, a specific IgA serum response to norovirus was observed in 70% of the Baylor subjects. These results indicate that norovirus VLPs are highly safe and effective in preventing viral infections [74,75]. Parvovirus B19 is a significant human pathogen that causes erythema infectiosum, also known as the fifth disease, a rash illness in children that can lead to arthralgia syndrome in adults [76]. Meridian Life Science (Memphis, TN, USA), Inc. has expressed VP1 and VP2 proteins using the BEVS platform, assembled these two capsid proteins into VLPs, and designated the product VAI-VP705. After the second dose, most vaccinated individuals produced ELISA and neutralizing antibodies against Parvovirus B19. Currently, VAI-VP705 is in clinical phase I/II trials (NCT00379938) [76]. The Ebola virus (EBOV) is a zoonotic illness that can lead to severe hemorrhagic fever and high mortality rates [77]. Novavax has infected Sf9 cells with recombinant baculovirus expressing EBOV/Mak GP, prepared EBOV/Mak GP nanoparticles, and combined them with Matrix-M to create a vaccine. In a mouse model, experimental results have shown that EBOV/Mak GP combined with the Matrix-M adjuvant induces high levels of antigen-specific IgG antibodies. Currently, the vaccine is undergoing clinical phase I trials (NCT02370589) [78].

Human respiratory syncytial virus (RSV) is a global pathogen that is the primary viral cause of severe lower respiratory tract disease in infants worldwide. More severe diseases occur in the elderly, immunocompromised patients, and patients with underlying cardiopulmonary diseases [79,80]. Novavax has developed a novel RSV F nanoparticle vaccine based on a purified, recombinant, near-full-length RSV fusion (F) glycoprotein [81]. This vaccine has a rapid vaccine-induced immune response that rises seven days after inoculation, with a peak level of anti-F protein IgG antibody from 3.6-fold to 5.6-fold compared to that of IgG elicited by natural RSV infection. The anti-F response persists 12 months after vaccination, and the neutralizing antibody after vaccination rises from 1.3-fold to 1.7-fold of the neutralizing antibody level elicited by natural RSV infection. The vaccine is currently in clinical phase III trials (NCT02624947) and has been shown to induce increased functional immunity to RSV in older adults with good safety [82]. In addition, Novavax’s seasonal influenza vaccine, NanoFlu, produced using the BEVS platform, is also in clinical phase III trials (NCT04120194). The H1N1 influenza vaccine, also manufactured by Novavax, is now in clinical phase II trials (NCT01072799). SinoCellTech (Beijing, China) has developed an HPV vaccine that is currently in clinical phase II trials (NCT05060484).

The BEVS platform can also be used to prepare adeno-associated virus (AAV) vaccines, which utilize baculovirus to infect insect cells Sf9, thereby simplifying virus vector production and reducing costs. Malaria is one of the primary infectious diseases leading to death worldwide caused by plasmodium infection [83]. Novavax’s ChAd63-MVA ME-TRAP vaccine was the first vaccine used in humans in a phase I clinical controlled study (NCT01669512). In this study, healthy adults showed good tolerance to intramuscular injections, with minimal local and systemic adverse reactions. T-cell ELISpot response peaked seven days post-boost vaccination, with MVA ME-TRAP and TRAP-specific IgG response highest at 28 days after boost vaccination [84].

### 5.3. BEVS-Derived Preclinical Vaccines

Many BEVS-derived recombinant protein vaccines are currently in the early development phase as candidates for different human diseases, including infectious diseases, diabetes, parasitic diseases, and tumors [85]. Considering a series of viruses belonging to *Flavivirus* in *Flaviviridae* as examples, the Zika virus (ZIKV) can cause severe neurological diseases, such as fetal microcephaly and Guillain–Barre syndrome. The envelope (E) protein of ZIKV is the primary target of vaccine research. A vaccine displaying ZIKV E protein on its surface has been derived from the AcMNPV recombinant baculovirus vector. Specific antibodies against ZIKV were produced in mice after immunization, effectively neutralizing ZIKV [86]. The BEVS-derived vaccine in the preclinical phase for yellow fever transmitted by the *Aedes mosquito* used tandem epitopes of E and NS1 proteins, eliciting low but significant neutralizing antibodies [87]. Furthermore, the West Nile virus vaccine is also in the preclinical phase. Bonafé et al. used recombinant West Nile virus truncated envelope protein antigen (rWNV-E) produced by the expresSF+^®^ insect cell line as a vaccine antigen. They found that the West Nile virus neutralization titer was induced in foals for at least 14 weeks, and no treatment-related clinical adverse reactions were detected in high-dose vaccinated rats [88]. Dengue fever, a prevalent acute infectious disease in Indonesia and other tropical countries, has four serotypes [89]. The E protein gene is the primary target of most BEVS model vaccines developed for these four serotypes. Jin Sun et al. designed two vaccines, cE80 (D4) and cE80 (max), which can stimulate specific antibodies against all four medium Dengue virus (DENV) serotypes in mice, mainly activating IgG1. In addition, they can activate type I and type II antigen-specific helper T cells that secrete IFN-γ and IL-4, respectively, which are currently in the preclinical phase [90,91]. In addition, many BEVS-derived vaccines for SARS-CoV-2 variants are in the preclinical phase.

## 6. Conclusions and Perspectives

The baculovirus expression vector system (BEVS) platform was established in the 1980s. After four decades of development, the BEVS has transitioned from a research tool to a mature manufacturing platform for producing new biological products, particularly vaccines. BEVS has been used to successfully express and purify thousands of proteins. The advantages of high safety and cost-effectiveness have made BEVS a competitive commercial manufacturing platform for vaccines and gene therapy vectors. Currently, vaccines produced using BEVS have been successfully marketed for the influenza virus, HPV, SARS-CoV-2, and some animal viruses. The availability of approved BEVS-derived vaccines removes hurdles for future product approval, providing regulator confidence. Many BEVS-derived clinical and preclinical vaccines also demonstrate the acceptance and productivity of this platform.

Although BEVS has some limitations, various strategies have been developed to generate recombinant viruses and improve recombinant proteins’ stability, yield, and post-translational modification. For instance, engineering insect cell lines has addressed the consideration that insect cells cannot produce proteins with complex N-glycans bearing terminal sialic acids like mammalian cells. In addition, optimizing the original vectors, leveraging gene editing technologies, and utilizing RNAi can delay cell apoptosis and enhance the stability of recombinant proteins, thus boosting their yield of recombinant proteins. In the future, improvements in the BEVS platform and downstream purification processes will allow BEVS to become more productive and less costly, the applications of BEVS platforms in vaccine development and gene therapy will be further promoted, and more applications based on the BEVS platform will be developed.

## Figures and Tables

**Table 1:** Strategies for optimizing BEVS.

Strategies	Designation		Optimization Purpose
Engineering of the baculovirus vector	Promoters:	orf46, pH, p10	Additive effect when combined
	Promoters:	39k, gp64	Contribute to protein expression
	Promoters:	OpIE2, IE1	As effective initiators of foreign gene expression
	Enhancers:	hr5, BSS, vp39	Enhance the activation ability of the pH promoter
	Enhancers:	hr1	Promote the protein expression
	Enhancers:	p19	Act as an RNAi inhibitor
	Remove non-essential genes	p10, p24	Design baculovirus vectors lacking chitinase and cathepsin
	Add beneficial genes to the viral genome	p35	Produce higher levels of recombinant proteins
Engineering of the cellular host	RNA interference apoptosis-related gene	p35, p49	Extend the expression of recombinant protein
	Establish cell lines	derived from Hi5, BmN, Sf9	Extend the expression of recombinant protein
	Classical insect cell lines	SF21, Sf9, Hi5, Tn-368	Produce less complex N-glycans and baculovirus infection leads to cell death or lysis
	Commercial insect cell lines	SfSWT-1, SfSWT-5	Produce highly processed recombinant proteins with terminal sialic acids complex type N-glycans

**Table 2:** BEVS-derived commercial vaccines.

Applicable Categories	Target	Name	Antigen	Product Type	Manufacture	Recommended Administration Schedule
Human vaccines	Influenza virus	Flublok^®^	HA protein	Subunit	Sanofi Pasteur (Paris, France)	Each year
	Influenza virus	Flublok Quadrivalent^®^	HA protein	Subunit	Sanofi Pasteur	Each year
	Papillomavirus	Cervarix™	HPV16/18 L1 protein	VLP	GSK (London, UK)	Three times in six months
	SARS-CoV-2	NVX-CoV2373	S protein	Subunit	Novavax (Malvern, PA, USA)	Eight weeks apart, two injections
	SARS-CoV-2	Weikexin	Recombinant RBD monomer	Subunit	Westvac (Chengdu, China)	Six months apart, two booster injections
	SARS-CoV-2	Trivalent Weikexin	Recombinant RBD monomer	Subunit	Westvac (Chengdu, China)	/
	SARS-CoV-2	VidPrevtyn Beta	SARS-CoV-2 preS dTM	Subunit	Sanofi/GSK (Paris, France/London, UK)	Four months
	SARS-CoV-2	SpikoGen^®^	S protein extracellular domain	Subunit	Vaxine/CinnaGen Co. (Adelaide, Australia/Tehran, Iran)	Three weeks apart
Animal vaccines	Classical swine fever	Porcilis^®^ Pesti	E2 protein	Subunit	MSD Animal Health (Shanghai, China)	Four weeks apart, two injections
	Classical swine fever	BAYOVAC CSF E2^®^	E2 protein	Subunit	Bayer AG/Pfizer Animal (Nordrhein-Westfalen, Germany/Groton, CT, USA)	Four to six weeks apart
	Porcine circovirus-2	CircoFLEX^®^	PCV2 ORF2 protein	VLP	B. Ingelheim (Berlin, Germany)	Piglets once, breeding pigs three times a year
	Porcine circovirus-2	Porcilis^®^ PCV	PCV2 ORF2 protein	VLP	MSD Animal Health	Two to three weeks apart, two injections
	Porcine circovirus-2	Circumvent^®^ PCV G2	PCV2a Cap protein	VLP	Merck Animal Health (Madison, NJ, USA)	Just one injection

**Table 3:** BEVS-derived clinical vaccines.

Target	Phase	Antigen	Product Type	Manufacture	NCT Number
Norwalk virus	Phase II	Norwalk virus-VLP	VLP	Baylor College of Medicine (Houston, TX, USA)	NCT00973284
	Phase I	Norwalk virus-VLP	VLP	LigoCyte (Bozeman, MT, USA)	NCT00806962
Parvovirus B19	Phase I/II	VP1 and VP2	VLP	Meridian Life Science (Memphis, TN, USA)	NCT00379938
Ebola virus	Phase I	EBOV Glycoprotein	Subunit	Novavax	NCT02370589
RSV	Phase III	Fusion glycoprotein	Nanoparticle	Novavax	NCT02624947
Malaria	Phase I	ChAd63-MVA ME-TRAP	Viral-vectored	Novavax	NCT01669512
Seasonal influenza virus	Phase III	HA, NA and M1	Nanoparticle	Novavax	NCT04120194
H1N1 influenza	Phase II	H1N1 2009 Influenza Virus-like Particle	VLP	Novavax	NCT01072799
Papillomavirus	Phase II	HPV (6/11/16/18/31/33/35/39/45/51/52/56/58/59) L1 protein	VLP	SinoCellTech (Beijing, China)	NCT05060484
SARS-CoV-2	Phase II	SARS-CoV-2 preS dTM	Subunit	Sanofi/GSK	NCT04762680
SARS-CoV-2	Phase I	SARS-CoV-2 S1 protein	VLP	Radboud University Medical Center (Nijmegen, Netherlands)	NCT04839146

## Data Availability

Not applicable.

## Abbreviations

BEVS	Baculovirus expression vector system
HPV	Human papillomavirus
AcMNPV	Autographa californica multicapsid nucleopolyhedrovirus
BmNPV	Bombyx mori nucleopolyhedrovirus
VLPs	Virus-like particles
RNAi	RNA interference
BSS	Burst sequences
hr1	Homologous region 1
sgMultiple	Multiple editing anti-BmNPV therapeutic complex CRISPR-Cas9 system, PSL1180-Cas9-sgIE1-sgLEF11-sgGP64
rBV	Recombinant baculovirus
RNAi	RNA interference
FDL	Fused lobed
rHA	Recombinant trivalent hemagglutinin
HA	Hemagglutinin
RIV4	Quadrivalent recombinant influenza vaccine
IIV4	Quadrivalent-inactivated influenza vaccines
hACE2	Human angiotensin-converting enzyme 2
S	spike
preS dTM	Prefusion transmembrane-deleted spike
M1	Matrix protein 1
EBOV	Ebola virus
RSV	Respiratory syncytial virus
F	Fusion
AAV	Adeno-associated virus
ZIKV	Zika virus
E	Envelope
rWNV-E	Recombinant West Nile virus truncated envelope protein antigen
DENV	Dengue virus
